# Vortioxetine monotherapy for treatment-resistant OCD: evidence from a retrospective multicentre study

**DOI:** 10.1192/j.eurpsy.2026.11010

**Published:** 2026-07-31

**Authors:** V. Martiadis, F. Raffone, S. Testa, C. Iaccarino, E. Pessina, A. Martini, T. Prodi, M. Olivola, C. I. Cattaneo, D. De Berardis, B. Benatti, B. M. Dell’Osso

**Affiliations:** 1 Department of Mental Health, Asl Napoli 1 Centro, Naples; 2 Department of Mental Health, Asl Cuneo 2, Bra; 3 Department of Biomedical and Clinical Sciences Luigi Sacco, Department of Psychiatry, ASST Fatebenefratelli-Sacco, University of Milan, Milan; 4 Department of Mental Health, Asl Biella, Naples; 5 Department of Mental Health, Asl Teramo, Teramo, Italy; 6 Department of Psychiatry and Behavioral Sciences, Bipolar Disorders Clinic, Stanford Medical School, Stanford University, Stanford, United States; 7 CRC ‘Aldo Ravelli’ for Neurotechnology & Experimental Brain Therapeutics; 8 Centro per lo Studio dei Meccanismi Molecolari alla base delle Patologie neuro-psico-geriatriche, University of Milan, Milan, Italy

## Abstract

**Introduction:**

Obsessive-compulsive disorder (OCD) resistant to selective serotonin reuptake inhibitors (SSRIs) remains a major clinical challenge, as many patients do not adequately respond to first-line treatments. Vortioxetine, a multimodal antidepressant approved for major depressive disorder, shows a favorable tolerability profile and potential cognitive benefits. However, evidence for its use in OCD is limited.

**Objectives:**

This study evaluated the efficacy and tolerability of vortioxetine monotherapy in adults with SSRI-resistant OCD.

**Methods:**

This retrospective, observational, multicentre study included 64 adults with OCD (DSM-5) who had not responded to at least one adequate SSRI trial (≥12 weeks, therapeutic dose). Patients received vortioxetine monotherapy (≥20 mg/day) for at least eight weeks. Symptom severity was measured using the Yale-Brown Obsessive-Compulsive Scale (Y-BOCS), Hamilton Depression Rating Scale (HAM-D), and Hamilton Anxiety Rating Scale (HAM-A) at baseline and weeks 2, 4, 6, and 8. Clinical response was defined as a ≥25% reduction in Y-BOCS. Adverse events were systematically recorded.

**Results:**

At week 8, 39.1% of patients achieved response. Mean Y-BOCS scores decreased from 27.1 to 20.7 (p<0.001), with improvements in both obsessions and compulsions. HAM-D and HAM-A scores also significantly decreased (p<0.001), from 21.0 to 12.6 and 26.9 to 16.1, respectively. Vortioxetine was generally well tolerated; 59.4% reported at least one side effect, most commonly nausea (29.7%). No serious adverse events were observed.

**Conclusions:**

These findings suggest vortioxetine monotherapy may be a promising and safe option for SSRI-resistant OCD. Controlled, prospective studies are needed to confirm its role in this population.

**Disclosure of Interest:**

None Declared

